# Traumatic injury clinical trial evaluating tranexamic acid in children (TIC-TOC): study protocol for a pilot randomized controlled trial

**DOI:** 10.1186/s13063-018-2974-z

**Published:** 2018-10-30

**Authors:** Daniel K. Nishijima, John VanBuren, Hilary A. Hewes, Sage R. Myers, Rachel M. Stanley, P. David Adelson, Sarah E. Barnhard, Matthew Bobinski, Simona Ghetti, James F. Holmes, Ian Roberts, Walton O. Schalick, Nam K. Tran, Leah S. Tzimenatos, J. Michael Dean, Nathan Kuppermann, for the TIC-TOC Collaborators of the Pediatric Emergency Care Applied Research Network, Marike Zwienenberg, Joseph Galante, Joseph Stephenson, Alfred F. Trappey, Jordan Sandhu, T. Charles Casper, Stephen Fenton, Doug Brockmeyer, Theodore Pysher, Michael L. Nance, Shih-Shan Lang Chen, Deborah Sesok-Pizzini, Raj Thakkar, Eric Sribnik, Kathleen Nichol

**Affiliations:** 1 Department of Emergency Medicine, UC Davis School of Medicine, 4150 V. Street, PSSB 2100, Sacramento, CA 95817 USA; 2 Department of Pediatrics, University of Utah School of Medicine, 295 Chipeta Way, Salt Lake City, UT 84108 USA; 3 Department of Pediatrics, Division of Pediatric Emergency Medicine, University of Utah School of Medicine, Primary Children’s Hospital, 100 N. Mario Capecchi Dr., Salt Lake City, UT 84113 USA; 4 Department of Pediatrics, Division of Pediatric Emergency Medicine, Perelman School of Medicine at the University of Pennsylvania, Children’s Hospital of Philadelphia, 3401 Civic Center Blvd., Philadelphia, PA 19104 USA; 5 Department of Pediatrics, Division of Pediatric Emergency Medicine, Ohio State University School of Medicine, Nationwide Children’s Hospital, 700 Children’s Dr., Columbus, OH 43205 USA; 6 Department of Pediatric Neurosciences, Barrow Neurological Institute at Phoenix Children’s Hospital, 1919 E. Thomas Rd, Phoenix, AZ 85016 USA; 7 Department of Pathology and Laboratory Medicine, UC Davis School of Medicine, 2315 Stockton Blvd., Sacramento, CA 95817 USA; 8 Department of Radiology, UC Davis School of Medicine, 2315 Stockton Blvd., Sacramento, CA 95817 USA; 9 Department of Psychology, University of California, Davis, 102K Young Hall, 1 Shields Ave., Davis, CA 95616 USA; 10 Department of Population Health, London School of Hygiene and Tropical Medicine, Keppel Street, London, WC1E 7HT UK; 11 Department of Orthopedics and Rehabilitation, University of Wisconsin, 317 Knutson Drive, Madison, WI 53704 USA; 12 Department of Pathology and Laboratory Medicine, University of California, Davis, 3422 Tupper Hall, Davis, CA 95616 USA; 13 Departments of Emergency Medicine and Pediatrics, UC Davis School of Medicine, 4150 V. Street, PSSB 2100, Sacramento, CA 95817 USA

**Keywords:** Children, Trauma, Tranexamic acid

## Abstract

**Background:**

Trauma is the leading cause of morbidity and mortality in children in the United States. The antifibrinolytic drug tranexamic acid (TXA) improves survival in adults with traumatic hemorrhage, however, the drug has not been evaluated in a clinical trial in severely injured children. We designed the Traumatic Injury Clinical Trial Evaluating Tranexamic Acid in Children (TIC-TOC) trial to evaluate the feasibility of conducting a confirmatory clinical trial that evaluates the effects of TXA in children with severe trauma and hemorrhagic injuries.

**Methods:**

Children with severe trauma and evidence of hemorrhagic torso or brain injuries will be randomized to one of three arms: (1) TXA dose A (15 mg/kg bolus dose over 20 min, followed by 2 mg/kg/hr infusion over 8 h), (2) TXA dose B (30 mg/kg bolus dose over 20 min, followed by 4 mg/kg/hr infusion over 8 h), or (3) placebo. We will use permuted-block randomization by injury type: hemorrhagic brain injury, hemorrhagic torso injury, and combined hemorrhagic brain and torso injury. The trial will be conducted at four pediatric Level I trauma centers. We will collect the following outcome measures: global functioning as measured by the Pediatric Quality of Life (PedsQL) and Pediatric Glasgow Outcome Scale Extended (GOS-E Peds), working memory (digit span test), total amount of blood products transfused in the initial 48 h, intracranial hemorrhage progression at 24 h, coagulation biomarkers, and adverse events (specifically thromboembolic events and seizures).

**Discussion:**

This multicenter trial will provide important preliminary data and assess the feasibility of conducting a confirmatory clinical trial that evaluates the benefits of TXA in children with severe trauma and hemorrhagic injuries to the torso and/or brain.

**Trial registration:**

ClinicalTrials.gov registration number: NCT02840097. Registered on 14 July 2016.

**Electronic supplementary material:**

The online version of this article (10.1186/s13063-018-2974-z) contains supplementary material, which is available to authorized users.

## Background

Trauma is the leading cause of morbidity and mortality in children in the United States [1]. Deaths from pediatric trauma are primarily from direct injury to critical organs (brain, heart, lungs) or hemorrhage into the thoracic and/or abdominal cavities. In the initial 24 h after injury, hemorrhage is the leading cause of death [2]. The degree of hemorrhage is influenced by both the extent of injury to the structures and the occurrence of trauma-induced coagulopathy. Traumatic coagulopathy is common after severe injury, particularly in patients with brain injuries, acidosis, and/or shock; it independently increases the risk of morbidity and mortality [3–6]. Nearly 30% of injured children admitted to the hospital have abnormalities of routine clotting parameters and 6% have markedly abnormal values [7].

Hyperfibrinolysis, or the premature and excessive breakdown of blood clots, is largely driven by increased plasmin generation after tissue injury and is a major component of traumatic coagulopathy [8, 9]. As measured by thromboelastography (TEG) testing, hyperfibrinolysis occurs in 24% of severely injured children and increases the risk for death sixfold compared to children without hyperfibrinolysis [10]. Hyperfibrinolysis is also associated with the need for lifesaving interventions and the need for blood product transfusions in severely injured children [11].

Tranexamic acid (TXA) is an antifibrinolytic lysine analog that inhibits plasminogen activation. Plasmin is central to fibrinolysis as it catalyzes the dissolution of fibrin clots. Premature and excessive fibrinolysis is a prominent characteristic of traumatic coagulopathy and hemorrhage progression due to elevated levels of tissue plasminogen activator (tPA) commonly seen after traumatic injuries, particularly traumatic brain injuries (TBI) [12].

Recent evidence in injured adults indicates that treatment with TXA decreases the risk of mortality following traumatic hemorrhage [13]. The Clinical Randomization of an Antifibrinolytic in Significant Haemorrhage-2 (CRASH-2) trial randomized 20,211 adult trauma patients at risk for significant hemorrhage to TXA or placebo. If administered within 3 h of injury, TXA reduced the risk of death from bleeding by approximately one third [14]. Furthermore, TXA had an excellent safety profile and was found to be cost-effective for use in these injured adult patients [15]. Based on the results of the CRASH-2 trial, administration of TXA is considered standard of care for severely injured adults with hemorrhagic trauma and is included on the World Health Organization’s (WHO) list of essential drugs.

A similar clinical trial has not been conducted in children with hemorrhagic injuries. We previously demonstrated that TXA is rarely given for injured children in US children’s hospitals [16]. Data regarding TXA use in children is mostly limited to those undergoing certain elective surgeries, specifically cardiac surgery [17–20], with some small studies in children undergoing spinal and craniofacial surgeries [17], and across a wide range of doses [17, 21]. These data suggest that perioperative administration of TXA decreases blood transfusion requirements in children. Furthermore, these studies suggest an excellent safety profile for TXA in children [17–20]. The type of traumatic insult and degree of inflammation and its impact on coagulation in patients with these elective surgeries, however, is likely different than that following hemorrhage from trauma.

Based on the TXA studies in injured adults and the evidence of safety and effectiveness in children undergoing non-traumatic surgical procedures, there is great potential that TXA may safely improve clinical outcomes in injured children. Not surprisingly, determining if TXA is beneficial and safe in injured children is a top priority for several stakeholders [6, 22–24]. To address this clinical question, we will conduct a pilot trial to demonstrate the ability to efficiently identify and enroll children with severe trauma and hemorrhagic injuries into a multicenter, randomized controlled trial evaluating two doses of TXA and placebo.

## Methods

### Study design

**Fig. 1:**
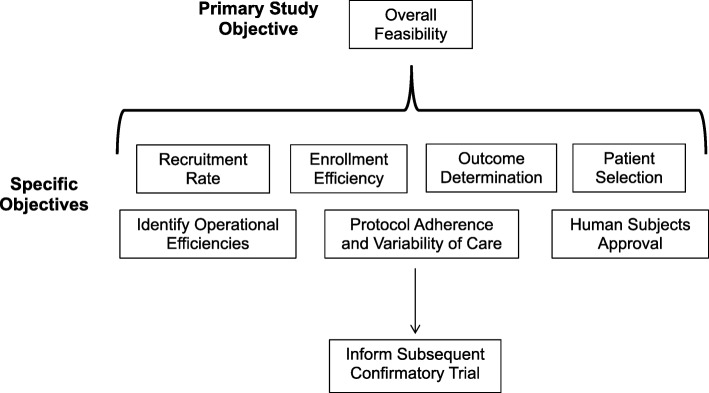
Study objectives of the TIC-TOC pilot trial

This pilot study is a double-blind, randomized, controlled trial of children younger than 18 years with hemorrhagic injuries to the torso and brain. We will enroll a maximum of 40 children during the study. Children will be randomized into one of three arms: (1) TXA dose A (15 mg/kg bolus dose over 20 min, followed by 2 mg/kg/hr infusion over 8 h), (2) TXA dose B (30 mg/kg bolus dose over 20 min, followed by 4 mg/kg/hr infusion over 8 h), and (3) normal saline placebo. This pilot study will be conducted in preparation for a confirmatory clinical trial of TXA administration for children with hemorrhagic torso and/or brain injuries (Fig. 1). SPIRIT (Standard Protocol Items: Recommendations for Interventional Trials) guidelines were followed and a checklist completed (Additional file 1) [25].

### Study setting

The pilot study will be conducted at 4 clinical sites. All sites are American College of Surgery (ACS) verified Level I pediatric trauma centers.

### Participants

#### Inclusion and exclusion criteria

Children younger than 18 years with evidence of hemorrhagic injuries to the torso and/or brain will be eligible. Eligible patients will be divided into three groups (head injury, torso injury, or head and torso injury) based on the inclusion criteria listed below. Patients in the combined head and torso injury group must meet entry criteria for both head and torso trauma. See Tables 1 and 2 for specific inclusion and exclusion criteria.

**Table 1:** Inclusion criteria for TIC-TOC trial

*Penetrating Torso Trauma:* • Penetrating trauma to the chest, abdomen, neck, pelvis, or thigh with at least one of the following: ◦ age-adjusted hypotension, or ◦ age-adjusted tachycardia despite adequate resuscitation fluids, or ◦ radiographic evidence of internal hemorrhage, or ◦ clinical suspicion of ongoing internal hemorrhage *Blunt Torso Trauma:* • Clinician suspicion of hemorrhagic blunt torso injury and at least one of the following: ◦ age-adjusted hypotension, or ◦ persistent age-adjusted tachycardia despite adequate resuscitation fluids • Hemothorax on chest tube placement or imaging; or • Clinical suspicion of hemorrhagic blunt torso injury *and* intraperitoneal fluid on abdominal ultrasonography (Focused Assessment with Sonography in Trauma; FAST); or • Intra-abdominal injury on CT with either contrast extravasation or more than trace intraperitoneal fluid; or • Pelvic fracture with contrast extravasation or hematoma on abdominal/pelvic CT scan with at least one of the following: ◦ age-adjusted tachycardia or ◦ age-adjusted hypotension *Head Trauma:* • GCS score 3 to 13 with associated intracranial hemorrhage on cranial CT scan	

*Abbreviations: CT* computed tomography, *GCS* Glasgow Coma Scale, *TOC-TOC* Traumatic Injury Clinical Trial Evaluating Tranexamic Acid in Children

**Table 2:** Exclusion criteria for TIC-TOC trial

Exclusion criteria include any of the below: 1. Unable to administer study drug within 3 h of traumatic event 2. Known pregnancy 3. Known prisoners 4. Known wards of the state 5. Cardiac arrest prior to randomization 6. GCS score of 3 with bilateral unresponsive pupils 7. Isolated subarachnoid hemorrhage, epidural hematoma, or diffuse axonal injury 8. Known bleeding/clotting disorders 9. Known seizure disorders 10. Known history of severe renal impairment 11. Unknown time of injury 12. Previous enrollment into the TIC-TOC trial 13. Prior TXA for current injury 14. Non-English and non-Spanish speaking 15. Known venous or arterial thrombosis	

*Abbreviations: GCS* Glasgow Coma Scale, *TOC-TOC* Traumatic Injury Clinical Trial Evaluating Tranexamic Acid in Children, *TXA* tranexamic acid

#### Participant screening and consent

**Fig. 2:**
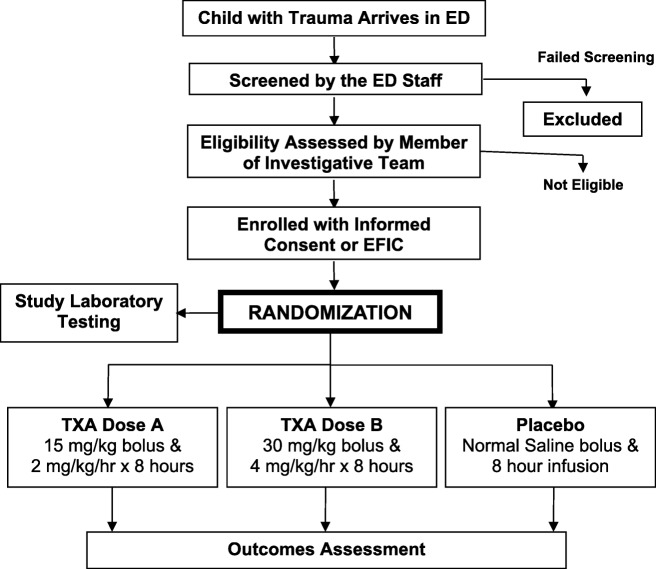
Study enrollment procedures. *Abbreviations*: *EFIC* exception from informed consent (enrollment without in emergency scenarios when prospective informed consent is not possible); *ED* emergency department; *TXA* tranexamic acid

Prior to the onset of the study and intermittently during its course, site clinical research coordinators and emergency department (ED) clinicians will be educated regarding the trial and trained to identify potentially eligible patients. Patients will be identified and recruited from the EDs of the participating centers. On arrival to the ED, all injured children will undergo primary and secondary surveys by the physicians providing clinical care in the ED. Injured children will then undergo diagnostic testing as deemed appropriate by the treating physicians (i.e., standard of care). These patients will be screened by clinical research coordinators and patients believed to be eligible will be discussed with the treating physicians. If the patient is believed to be eligible, the coordinator will contact the study site investigative team to discuss eligibility. A study investigator (a clinician, determined by each site) will evaluate the patient and determine eligibility (Fig. 2). At the start of this pilot trial, we enrolled patients only if written informed consent was obtained from parents or legal guardians. After starting the trial, however, it became evident that most eligible patients did not have a parent or legal guardian available to provide written informed consent at the time of ED presentation and study eligibility. We therefore revised the protocol to include Exception from Informed Consent (EFIC) procedures if the parent or legal guardian was not available to provide written informed consent at the time of study eligibility.

### Interventions

Enrolled patients will be randomized to one of three study arms: TXA dose A, TXA dose B, or placebo. A systematic review demonstrated substantial variability in TXA dosing for pediatric surgical patients, ranging from initial loading doses of 2 to 100 mg/kg intravenously (IV), and a continuous infusion ranging from 3 to 10 mg/kg/h [17]. Doses selected for this study and rationale are as follows:

- TXA dose A arm: Patients will receive a 15 mg/kg bolus of TXA over 20 min followed by a 2 mg/kg/h infusion over 8 h. The maximum bolus dose is 1000 mg, the maximum rate of infusion is 50 mg/min, and the maximum total maintenance dose is 1000 mg. This represents a total TXA dose of 31 mg/kg. This dosing is based on the CRASH-2 trial and has been recommended by a prior evidence statement [26]. This dose is also estimated to inhibit approximately 80% of fibrinolysis based on prior TXA studies [27].
- TXA dose B arm: Patients will receive a 30 mg/kg bolus of TXA over 20 min followed by a 4 mg/kg/h infusion over 8 h. The maximum bolus dose is 2000 mg, the maximum rate of infusion is 100 mg/min, and the maximum total maintenance dose is 2000 mg. This represents a total dose of TXA of 62 mg/kg. This represents approximately the 75th percentile dosage for children receiving TXA for non-traumatic surgical procedures [16] and is estimated to inhibit 100% of fibrinolysis [27]. This dose is also within the range recommended by the WHO [28] and has not demonstrated an increase in adverse events (compared to lower doses) [17].
- Placebo arm: Patients in the placebo group will receive a bolus dose of normal saline over 20 min followed by a normal saline infusion over 8 h (in the same weight- based volume as the other study arms).

The study drug will be discontinued if any of the following occur: suspected anaphylactic reaction, severe renal impairment (creatinine clearance less than 29 mL/min/1.73m^2^) is identified on subsequent laboratory measurements, withdrawal of consent by the patient’s legal guardian or legally authorized representative, or discovery of new information which makes the patient ineligible to continue participation in the study.

Both study patients and study team members are blinded to the interventional arm. Blinding is provided by the use of identical study drugs, packaging, volume, and rates of infusion. Unblinding will not be allowed as there is no reversal agent for TXA. Clinicians will be asked to assume the patient has received TXA and treat accordingly.

### Randomization

Due to the narrow window of efficacy of TXA based on the CRASH-2 trial, randomization must only minimally delay treatment. To complete the randomization quickly, the study intervention will be pre-assigned using a central randomization process. Prior to enrollment at each site, a study drug box containing a vial of masked study drug with a numeric identification code corresponding to the treatment assignment will be designated.

Because we will be evaluating TXA for different injury patterns (hemorrhagic torso injury, hemorrhagic brain injury, and combined hemorrhagic torso and brain injuries), randomization will be stratified by injury pattern. Eligible patients will be randomized into one of the three arms in a 1:1:1 ratio (TXA dose A, TXA dose B, or placebo). We will perform permuted-block randomization across injury pattern (i.e., torso or brain or both torso and brain). To ensure a sufficient number of injury types, we will limit the enrollment of patients meeting the inclusion criteria for isolated brain injury to 20 patients (as it is anticipated these will be the most common eligible patients evaluated at the participating sites). A patient is considered enrolled when randomization occurs.

### Outcomes

We will collect the following outcome data: the Pediatric Quality of Life Inventory (PedsQL) score, the Pediatric Glasgow Outcome Scale - Extended (GOS-E Peds) score, digit span recall (a test of working memory), total blood products (ml/kg) transfused over the initial 48 h of care (children with torso injuries), intracranial hemorrhage progression in first 24 h (children with brain injuries), coagulation biomarkers, and adverse events.

**Fig. 3:**
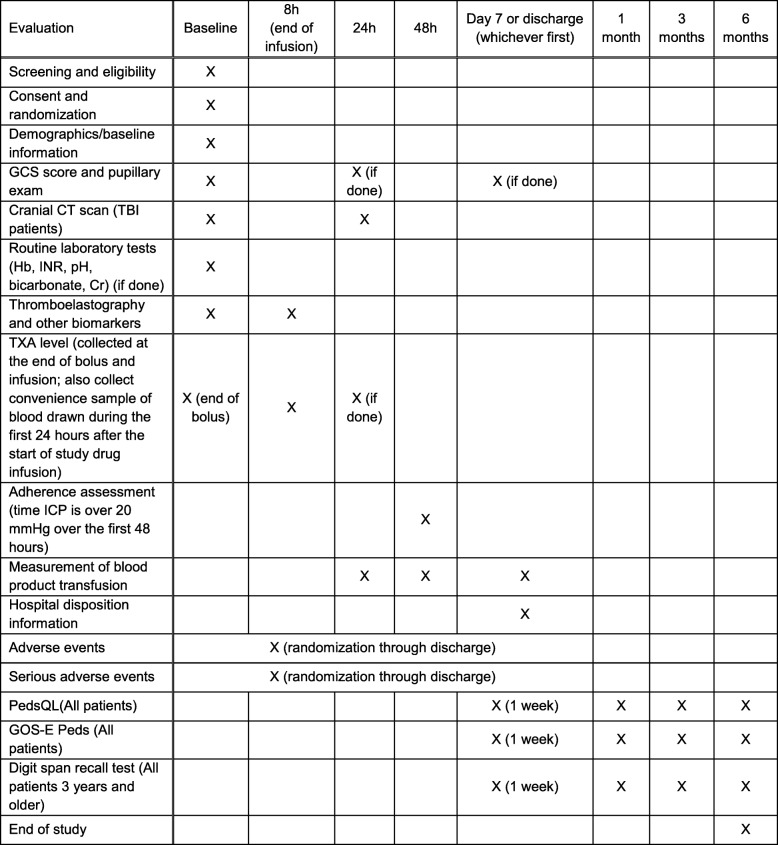
Schedule of evaluations. *Abbreviations*: *Cr* creatinine, *CT* computed tomography, *GCS* Glasgow Coma Scale, *GOS-E Peds* Pediatric Glasgow Outcome Scale – Extended, *Hb* hemoglobin, *ICP* intracranial pressure, *INR* international normalized ratio, *PedsQL* Pediatric Quality of Life, *TBI* traumatic brain injury, *TXA* tranexamic acid

We will assess neurocognitive functioning and other quality-of-life measures using the PedsQL and the GOS-E Peds 1 week, 1 month, 3 months, and 6 months after ED presentation for all enrolled children (Fig. 3). We will assess working memory using the digit span recall test 1 week, 1 month, 3 months, and 6 months after ED presentation for all enrolled children 3 years and older. Blood product transfusion will be calculated as the total ml/kg blood products received from randomization to 48 h after randomization. Blood products in the calculation will include red blood cell components, platelet components, plasma components, and cryoprecipitate. Prior to study initiation, collaborating trauma surgeons, transfusion medicine physicians, pediatric critical care physicians, pediatric anesthesiologists, and pediatric emergency medicine physicians will establish general guidelines for indications and thresholds for blood product transfusion. We will perform non-contrast cranial computed tomography (CT) scans 24 (± 6) hours after randomization to assess intracranial hemorrhage progression (for those who have not received a second CT scan during the specified time frame in the course of clinical care). We will exclude those who received a neurosurgical intervention from assessment of the 24 h CT scan. Additional brain-imaging studies may be performed at the discretion of the treating physician as a part of routine care. A study neuroradiologist, blinded to clinical data, will review cranial CT scans and calculate intracranial hemorrhage progression using the ABC/2 volume estimation [29]. Intracranial hemorrhage will be assessed relative to the total brain volume (calculated by the XYZ/2 volume estimation) [30, 31]. We will also measure coagulation biomarker tests before and after study drug infusion. These tests include: kaolin activated TEG (measuring fibrinolysis as the percentage of clot lysis at 30 min post-maximum clot strength), d-dimer, tissue plasminogen activator (tPA), plasmin-antiplasmin (PAP) complex, and plasmin generation. Parameters are performed at a central laboratory to eliminate inter-assay variability and to standardize testing. Safety outcomes will be assessed on the 7th day after randomization or at hospital discharge (whichever comes first) via review of the electronic medical record and include:

- Thromboembolic disease: any venous or arterial thrombosis on diagnostic imaging post-randomization
- Seizures occurring within the initial 24 h of study drug administration: clinical or electroencephalogram-documented

All study patients will be followed for 180 days after randomization or death, whichever comes first, regardless of whether or not the patient has completed the study intervention.

### Data management and safety monitoring plan

The study Data Coordinating Center (at the University of Utah) will create the electronic data capture system and worksheets. Data will be entered via the internet into the electronic data capture system. Worksheets and study documents will be maintained in locked file cabinets in locked offices at each site.

A trained site monitor will conduct site-monitoring visits during the study period to ensure regulatory compliance and patient safety, and to monitor the quality of data collected at each enrolling site. The site monitor will provide each site with a written report, and sites will be required to follow-up on any deficiencies.

The study will have a Data Safety Monitoring Board (DSMB) approved by the funding agency to advise the sponsors and principal investigators regarding the continuing safety of study patients and the continuing scientific merit of the study. The DSMB will have a charter, approve the protocol prior to implementation, and will review interim analyses as applicable. The DSMB is responsible for monitoring accrual of study patients, ensuring adherence to the study protocol, assessing data quality, reviewing the performance of individual clinical sites, and monitoring serious adverse events and other patient safety issues. The DSMB will include a biostatistician, an ethicist, a pediatric trauma surgeon, a pediatric neurosurgeon, a patient advocate, and an emergency medicine physician.

### Statistical considerations

As this study is a pilot trial to assess the feasibility of study procedures, the study is not powered for a particular endpoint. We estimate that there will be 1.25 eligible patients per site per month. This eligibility rate would justify the feasibility of conducting a subsequent confirmatory clinical trial evaluating the safety and efficacy of TXA in severely injured children. We will also evaluate the feasibility of enrolling patients with parent or legal guardian written informed consent only. If these consent procedures are not feasible (particularly given the 3 h from time of injury enrollment window and the frequent absence of parents/guardians at the time of ED presentation and study eligibility), we will apply to use EFIC procedures if parents or legal guardians are not available to provide written informed consent. We set a priori thresholds for recruitment futility to convert to the use of EFIC for patient enrollment. We will collect detailed information on missed eligible patients to evaluate the feasibility of consent processes to determine if and when to initiate EFIC procedures. Ultimately our a priori thresholds to convert to EFIC procedures were met. Our trial protocol was revised to include EFIC procedures if parents or guardians are not available or able to provide written informed consent.

## Discussion

The primary objective of this pilot trial is to assess the overall feasibility for a subsequent, confirmatory clinical trial evaluating the efficacy of TXA in children with severe hemorrhagic injuries. We designed the pilot trial to mirror closely the subsequent confirmatory trial regarding study design, interventional arms, procedures, and outcome measures. Real-world implementation of the study protocol will allow for assessment of various aspects of the pilot trial. This will provide critical information on the number of eligible patients at study sites, feasibility of timely consent and enrollment procedures, and protocol adherence and variability of care. Although we will not use the results of this pilot trial to estimate sample size for the subsequent confirmatory trial, we will assess the feasibility of obtaining various outcome measurements to help inform the subsequent trial.

Clinical trials of critically ill children are typically more challenging compared to similar trials in adults. Pediatric trials draw from a smaller patient pool compared to adult trials, making it more difficult to recruit adequate numbers of subjects [32]. Issues regarding parental consent and child assent often lead to variability in consent processes and local ethic committee reviews [32, 33]. The many administrative tasks such as Institutional Review Board (IRB) approvals and consortium agreements can delay the start of projects and increase costs [34–36]. These challenges have led to an inadequate number of pediatric clinical trials [19]. Specifically for the evaluation of TXA in trauma, there are currently 10 completed or open clinical trials in adults with hemorrhagic trauma but none in children [37].

Although specific strategies have been recommended to improve the efficiency of clinical trials, it is often difficult for investigators to identify all the potential obstacles to optimize trial efficiency before actually starting the trial [38–40]. Premature closure of phase III clinical trials due to insufficient patient accrual wastes substantial time and resources [41, 42]. Pilot trials, designed to enroll a small number of patients with the primary goal of assessing the feasibility of a subsequent phase III trial, provide a less expensive alternative to embarking on a costly, large-scale trial [43]. Pilot studies conducted prior to definitive studies are particularly crucial in clinical trials of critically ill children, which by nature are at risk for low patient accrual and complex regulatory requirements.

Previous pediatric pilot studies have provided clear answers regarding the feasibility of subsequent phase III trials. The Thrombolysis in Pediatric Stroke (TIPS) trial was a phase I trial funded by the National Institute for Neurological Disorders and Stroke (NINDS) to assess treatment with intravenous tPA in children with acute ischemic stroke [32]. Despite more than 200 meetings and 3 years of preparation, the trial was prematurely stopped due to lack of patient accrual. Only one patient was enrolled in the 17 active sites (median time active, 9 months). Other issues identified in the trial included delays for human subjects approval, costs for tPA storage, and availability of tPA at study site pharmacies. In contrast, the Therapeutic Hypothermia After Pediatric Cardiac Arrest (THAPCA) trial conducted a pilot phase to assess the feasibility of the larger scale trial [44]. The National Heart, Lung, and Blood Institute (NHLBI) recommended an 18-month pilot phase prior to two larger trials (one for in-hospital cardiac arrest and one for out-of-hospital cardiac arrest) due to concerns regarding the complexities of the trials, proposed costs, the feasibility of the consent process, and the ability to obtain long-term follow-up in critically ill children [44]. Ultimately, the pilot phase demonstrated successful recruitment (threshold attained 4 months ahead of schedule) and included four protocol amendments to augment enrollment. The full-scale THAPCA trials were subsequently approved and completed [45].

We acknowledge certain limitations of our pilot trial. We initially designed this trial using federal EFIC procedures; however, we were advised by the Food and Drug Administration (FDA) to initiate the pilot trial enrolling only with written informed consent. After the pilot study started, it became evident that most eligible patients did not have parents or legal guardians available to provide written informed consent. The FDA subsequently allowed EFIC procedures if eligible patients did not have parents or legal guardians available to provide written informed consent at the time of ED presentation and study eligibility. The unavailability of parents or legal guardians to provide written informed consent for injured children in our trial is consistent with prior literature. Approximately half of children with moderate-to-severe TBI do not have their guardian present in the ED in the first 3 h after injury [46]. Future work is planned to qualitatively and quantitatively evaluate parent or legal guardian perspectives regarding informed consent procedures for enrolling severely injured children into clinical trials. Another potential limitation is that the results of the pilot trial may not be generalizable to a much larger number of clinical sites for the subsequent confirmatory study. However, because the pilot trial is being conducted at four clinical sites, with each site bringing its own demographic, geographic, and clinical diversity to the trial, we feel that we will be able to better anticipate potential barriers for the subsequent trial. Moreover, prior to the start of the pilot trial, we will have established general guidelines for blood transfusion and neurosurgical management that will be standardized across clinical sites.

In conclusion, this pilot trial will be the first randomized clinical trial to evaluate TXA in children with severe trauma and hemorrhagic injuries. The results of this pilot trial will provide crucial preliminary information to conduct a confirmatory trial.

### Trial status

This manuscript is based on protocol version 2.03 (August 22, 2018). The trial opened for enrollment in July 2018 and we anticipate the trial will be completed by December 2019.

## Additional file

Additional file 1: SPIRIT 2013 Checklist: recommended items to address in a clinical trial protocol and related documents. (DOCX 63 kb)

## Abbreviations

ACS: American College of Surgery
CRASH-2: Clinical Randomization of an Antifibrinolytic in Significant Haemorrhage
CT: Computed tomography
DSMB: Data Safety Monitoring Board
ED: Emergency department
EFIC: Exception from Informed Consent
EMSC: Emergency Medical Services for Children
FDA: Food and Drug Administration
GOS-E Peds: Pediatric Glasgow Outcome Scale - Extended
HHS: Health and Human Services
HRSA: Health Resources and Services Administration
IRB: Institutional Review Board
IV: Intravenous
MCHB: Maternal and Child Health Bureau
NCATS: National Center for Advancing Translational Sciences
NHLBI: National Heart, Lung, and Blood Institute
NINDS: National Institute for Neurological Disorders and Stroke
PAP: Plasmin-antiplasmin
PedsQL: Pediatric Quality of Life
TBI: Traumatic brain injury
TEG: Thromboelastography
THAPCA: Therapeutic Hypothermia After Pediatric Cardiac Arrest
TIC-TOC: Traumatic Injury Clinical Trial Evaluating Tranexamic Acid in Children
TIPS: Thrombolysis in Pediatric Stroke
tPA: Tissue plasminogen activator
TXA: Tranexamic acid
WHO: World Health Organization
